# Evaluation of D-TACE combined with endovascular brachytherapy for HCC with MPVTT

**DOI:** 10.3389/fonc.2022.973357

**Published:** 2022-08-18

**Authors:** Wei Huang, Ju Gong, Qingbing Wang, Ziyin Wang, Qin Liu, Jingjing Liu, Junwei Gu, Xiaoyi Ding, Zhiyuan Wu

**Affiliations:** ^1^ Department of Interventional Radiology, Ruijin Hospital, Shanghai Jiao Tong University School of Medicine, Shanghai, China; ^2^ Department of Interventional Radiology, Ruijin Hospital Luwan Branch, Shanghai Jiao Tong University School of Medicine, Shanghai, China

**Keywords:** hepatocellular carcinoma, portal vein, stents, endovascular brachytherapy, chemoembolization, doxorubicin-eluting beads

## Abstract

**Background:**

Hepatocellular carcinoma (HCC) patients with main portal vein tumor thrombus (MPVTT) may be able to have TACE through stent implantation into the portal vein with thrombosis to recover portal blood flow.

**Purpose:**

The goal of this study was to compare clinical results of conventional transcatheter arterial chemoembolization (C-TACE) and doxorubicin-eluting bead transcatheter arterial chemoembolization (D-TACE) combined with endovascular brachytherapy in HCC patients with MPVTT.

**Methods:**

This study was a retrospective controlled study with follow-up dates spanning from Mar 2015 to Feb 2020. Patients with both HCC and MPVTT were divided into two groups. Portal vein stents with iodine-125 seed strands were implanted first; then, C-TACE or D-TACE was administered to all patients. Objective response rates were assessed.

**Results:**

A total of 26 patients were enrolled, with 13 in each group. During follow-up, the portal stent patency times were 112.3 ± 98.2 days in the C-TACE group and 101.7 ± 90.4 days in the D-TACE group. The time to disease progression was 42 days in the C-TACE group and 120 days in the D-TACE group (p=0.03). The overall survival time from the first intervention procedure was 216 days in the C-TACE group and 239 days in the D-TACE group (p=0.047). The D-TACE group was superior to the C-TACE group in terms of progression-free survival (PFS) and overall survival (OS) times.

**Conclusion:**

Endovascular implantation of brachytherapy combined with TACE is safe and effective in HCC patients with MPVTT. This combination therapy may be helpful for survival benefits to patients with stage BCLC-C HCC.

## Background

Hepatocellular carcinoma (HCC) is a common malignant tumor. HCC patients with main portal vein tumor thrombus (MPVTT) often miss the opportunity for transcatheter arterial chemoembolization (TACE), a preferred nonsurgical option for liver cancer, because MPVTT is a relative contraindication for TACE. Previous studies have demonstrated that this type of patient may be able to have TACE through stent implantation into the portal vein with thrombosis to recover portal blood flow (1). The implantation of a radioactive seedling tent into the portal vein stent combined with TACE and MPVTT increases the stent patency rate and survival time (2, 3). Conventional TACE (C-TACE) embolic materials are always based on iodinated oil. A recent study reported the application of new embolic material loaded with doxorubicin class drug-eluting beads (DEB). Preliminary results show that the efficacy of DEB-TACE (D-TACE) in HCC is good and has low toxicity (4). However, whether D-TACE has greater survival benefits is still controversial (5). A DEB product, DC Bead™ (Biocompatibles UK Ltd.), has been officially approved by the China Food and Drug Administration and launched in China. However, few studies have examined the effects of radioactive seed stent implantation in a portal vein stent combined with D-TACE.

This preliminary study aimed to investigate the feasibility and safety of implanting a radioactive seed stent in a portal vein stent combined with TACE in HCC patients with MPVTT, and it compared the clinical effects of combined therapies with C-TACE or D-TACE.

## Methods

### Subjects

In this retrospective controlled study, 26 HCC patients with MPVTT were consecutively enrolled from March 1, 2015, to August 31, 2019 with subsequent follow-ups until February 29, 2020.

The included patients met the following conditions: age 18 to 80 years; diagnosis met the pathological or clinical diagnostic criteria of HCC; CT or MRI imaging showed that portal vein thrombosis involved the portal vein trunk and primary branch, but that the contralateral primary branch was not completely occluded; the liver function stage was Child-Pugh class A/B; patients had no extensive extrahepatic metastases; and the Eastern Cooperative Oncology Group (ECOG) score of the patient was 0–2. Due to economic constraints or concerns about the side effects of targeted drugs, these patients were not able to combine targeted drugs at the same time. All patients signed informed consent forms for this study.

Patients were excluded for the following reasons: the liver function stage was Child-Pugh class C; the patient had other serious diseases and could not complete treatment; or the patient had bleeding tendency with elongated coagulation time.

### Intervention procedures

All patients underwent radioactive seeding in the portal vein immediately followed by C-TACE or D-TACE. First, under the guidance of ultrasound, the portal vein branch of the uninvolved liver lobe was percutaneously punctured and a vascular sheath was inserted. Second, angiography was performed over the main portal vein stenosis segment with a 4F pigtail catheter (Cordis, USA), and the pressure was measured. The diameter and length of the stent as well as the number of required iodine-125 (^125^I) seeds were based on the stenosis segment length. The stents should extend 1 cm beyond each end of the tumor thrombus. The number of ^125^I seeds (0.6 mCi/tablets, Shanghai Xinke Pharmaceutical Co., Ltd.) were calculated using the formula [stenosis length (mm)/4.5+2] to ensure that the radiation range of implanted ^125^I seeds completely covered the portal vein thrombosis segment. The required ^125^I seedlings were encapsulated in a 3F sterile sheath and developed into a seed strand. Third, the stents and the ^125^I seed strips were placed into the portal vein vessels of the stenosis segments under the guidance of the X-ray perspective. Fourth, portal vein angiography was performed with the pigtail catheter; the pressure at this time was measured again. The liver puncture channel was blocked with a 5 mm × 5 cm coil (Cook Company, USA).

In the C-TACE group, the lipiodol dosage was based on lesion size. A tumor with a diameter of 1 cm corresponded to 1 ml of lipiodol with a maximum dose of 20 ml. Epirubicin (40 mg, Pharmorubicin, Pfizer) was mixed with the lipiodol, forming an emulsion. In the D-TACE group, the doxorubicin-eluting (DC) bead diameters were 300 μm to 500 μm. One to two bottles were used based on lesion size, each containing 40 mg epirubicin. After combining the epirubicin with DC beads, a nonionic contrast agent iopamidol injection (370 mg I/mL) was mixed at a 1:1 proportion. TACE was performed using the femoral artery approach. The abovementioned embolic agents were slowly injected into the tumor-feeding artery for embolization after superselective catheterization. Gelatin sponges were added to strengthen the embolism.

Patients received liver protection treatment with symptomatic and supportive treatment for 7 to 8 days after interventional procedures. Blood tests, liver renal function tests, and electrolytes were measured at 3 days, 7 days, 14 days and 30 days after the procedures. Complications were recorded and treated accordingly. Follow-up was performed every 3 months after the initial treatment. The clinical results mainly includes the changes of liver function, complications, the time to disease progression (estimated using enhanced abdominal CT or abdominal MRI) and survival rate. Objective response rates were assessed, and TACE treatments were performed as needed. The patency of the portal stent was compared between the two groups. The median progression-free survival (PFS) and overall survival (OS) times were assessed after long-term follow-up.

### Statistical analyses

The statistical analyses were performed using SPSS statistical software, version 23 (IBM, Armonk, NY, USA). A paired t-test was used to compare the changes in portal vein pressure before and after stent implantation. The Mann-Whitney test was used to compare the liver function and stent patency of the two groups. Progression-free survival and overall survival were analyzed with Kaplan–Meier and log-rank tests. All data are expressed as the mean ± SEM (standard error of the mean) of n independent measurements. GraphPad Prism 7 software (GraphPad, San Diego, CA, USA) was used to plot the graphs. A value of P < 0.05 was considered as statistically significant.

## Results

### Patients characteristics

Twenty-six patients (aged 40-78 years) were included in the analysis. **Table 1** shows the patients’ general information. The median age of the C-TACE group (9 men and 4 women) was 54.5 years; in the D-TACE group (11 men and 2 women), the median age was 56 years. Based on BCLC staging criteria, the all the patients were in stage C. Based on the Child-Pugh classification standard of liver function, in the C-TACE group, 9 cases were Child-Pugh class A, the other 4 cases were class B, and the mean Child-Pugh score was 6.15 ± 0.90. In the D-TACE group, 11 cases were Child-Pugh class A, the other 2 cases were class B, and the mean Child-Pugh score was 5.77 ± 0.73. No significant differences were found between the two groups (Mann-Whitney U=64.5, p=0.31). All patients had HCC lesions. In the C-TACE group, there were 4 cases in the left liver and 9 cases in the right liver. In the D-TACE group, there was one case in the left liver and all other cases were in the right liver. All the patients had tumor-side portal vein branch thrombus and MPVTT. Among these, 6 cases were accompanied by contralateral first level portal vein branch tumor thrombi in the C-TACE group and 4 cases were accompanied by contralateral first level portal vein branch tumor thrombi in the D-TACE group. There was no significant difference in post embolization syndrome between two groups.

**Table 1:** General information.

		C-TACE group	D-TACE group
Number of cases		13	13
Male: female		9:4	11:2
Average age (years)		54.5 ± 9.9	56.0 ± 9.2
CPC	A	9 (69.2%)	11 (84.6%)
	B	4 (30.8%)	2 (15.4%)
Primary tumor	left lobe: right lobe	4:9	1:10
	length (cm)	11.2 ± 1.3	10.0 ± 1.1
Other metastasis		none	none
VP class		VP 4 (100%)	VP 4 (100%)

CPC, Child-Pugh class; VP class, portal vein tumor thrombosis classification according to the Liver Cancer Study Group of Japan

### Effect of interventional therapy

**Figure 1:**
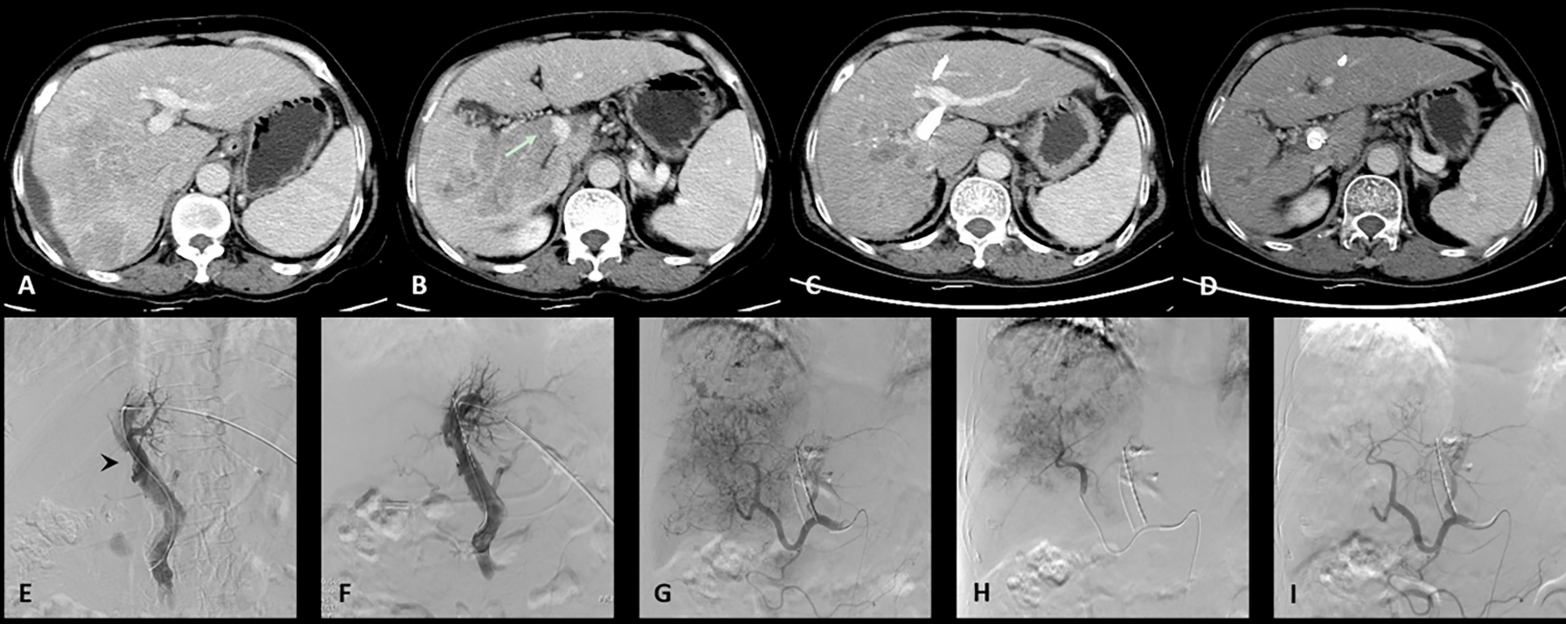
One case of the C-TACE group. The patient was a 60 years old female. **(A)**: Preoperative CT images showed a huge tumor in the right lobe of the liver. **(B)**: Tumor thrombus (arrow) was seen in the right branch and main portal vein. **(C)**: After 3 months follow up, the tumor was significantly reduced. **(D)**: The patency of portal vein stent was revealed after 3 months follow up. **(E)**: Filling defect was showed in the main portal vein (arrow head) during venography. **(F)**: Portal vein stent (diameter: 12 mm, length: 60 mm) was implanted and 12 125I radioactive seeds were used. **(G)**: Hepatic artery angiography showed large tumor staining of the right lobe of the liver. **(H)**: Superselective embolization of the tumor artery branches with 50 milligrams of epirubicin mixed with 10 ml lipiodol and 1 box of gelatin sponges was used to enhance embolization. **(I)**: Angiography after embolization, tumor blood supply was significantly reduced.

**Figure 2:**
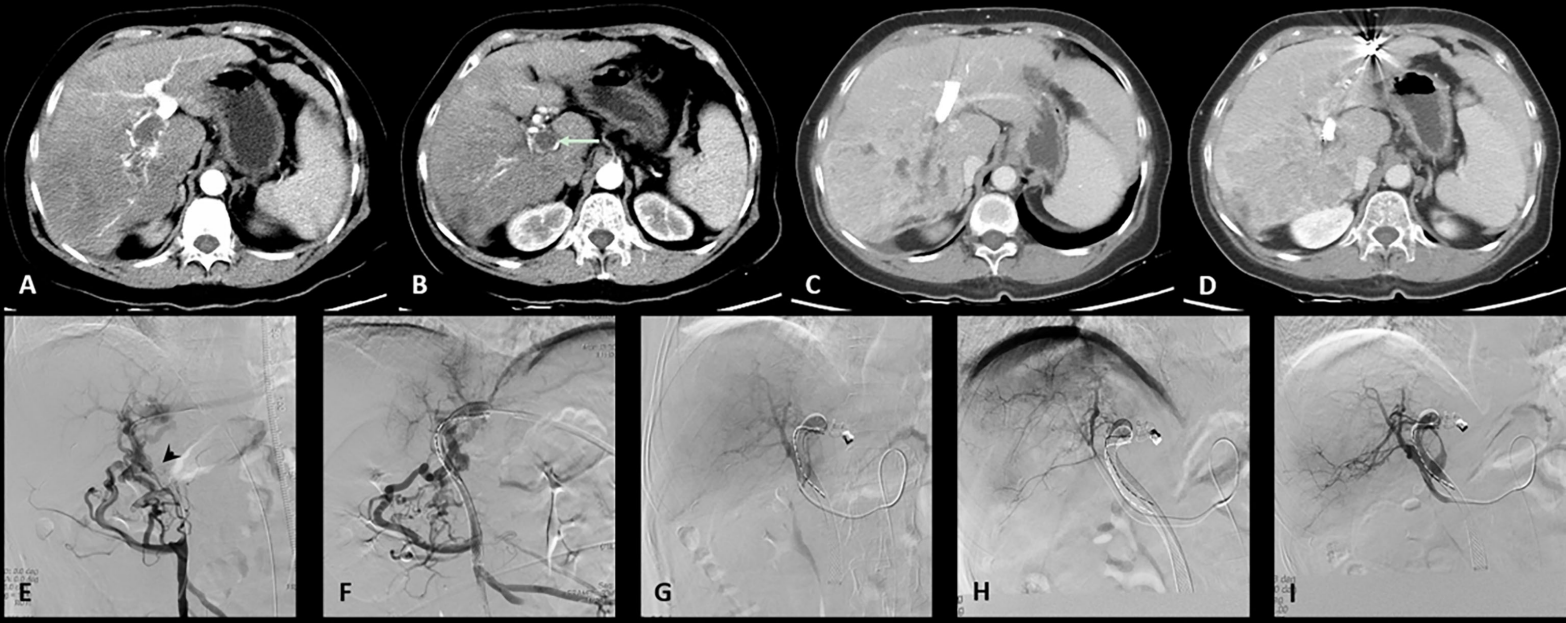
One case of the D-TACE group. The patient was a 69 years old female. **(A)**: Preoperative CT images showed a huge tumor in the right lobe of the liver. **(B)**: Tumor thrombus (arrow) was seen in the right branch and main portal vein. **(C)**: After 3 months follow up, the tumor was significantly reduced. **(D)**: The patency of portal vein stent was revealed after 3 months follow up. **(E)**: Filling defect was showed in the main portal vein (arrow head) during venography. **(F)**: Portal vein stent (diameter: 10 mm, length: 94 mm) was implanted and 20 125I radioactive seeds were used. **(G)**: Hepatic artery angiography showed large tumor staining of the right lobe of the liver. **(H)**: Superselective embolization of the tumor artery branches with 40 milligrams of epirubicin mixed with one bottle of DC beads. **(I)**: Angiography after embolization, tumor blood supply was significantly reduced.

All the patients successfully underwent stent implantation of a radioactive seed into the portal vein followed by TACE treatment. In the C-TACE group (**Figure 1**), 13 stents were implanted (diameter: 8-14 mm, length: 60–90 mm), 13^125^I radioactive seeds were used (a total of 204^125^I seeds, with an average of 16 per strip), and 10.6 ml of lipiodol was used in each case on average. Fifty milligrams of epirubicin were mixed with lipiodol for each case, and 15 boxes of gelatin sponges were used to enhance embolization. In the D-TACE group (**Figure 2**), 13 stents were implanted (diameter: 8-14 mm, length: 40-94 mm), and 13^125^ I radioactive seeds were used (a total of 181^125^I seeds, with an average of 14 per stripe); 18 bottles of DC beads were used; and five boxes of gelatin sponges were used to enhance embolization. Each bottle of DC beads contained a mixture of 40 mg of epirubicin.

Intraoperative angiography of the portal vein showed that the portal vein length of the tumor thrombus was 25.3 to 95.2 mm (average: 46.6 ± 19.1 mm) in the C-TACE group and 17.6 to 65.7 mm (average: 38.8 ± 16.5 mm) in the D-TACE group. In the C-TACE group, the average pressure of the distal main portal vein was 28.3 ± 11.2 cmH_2_O and 23.6 ± 10.2 cmH2O before and after stent implantation, respectively, a decrease of 4.6 ± 3.0 cmH_2_O, but the difference before and after stent implantation was not statistically significant (U=74.5, P=0.62). In the D-TACE group, the average pressures were 25.2 ± 12.3 cmH_2_O and 20.2 ± 11.7 cmH_2_O, respectively, a decrease of 5.0 ± 4.4 cmH2O, which was also not statistically significant (U=62.5, P=0.27). The difference in the magnitude of pressure decrease between the two groups was not statistically significant (U=74.5, P=0.62).

**Figure 3:**
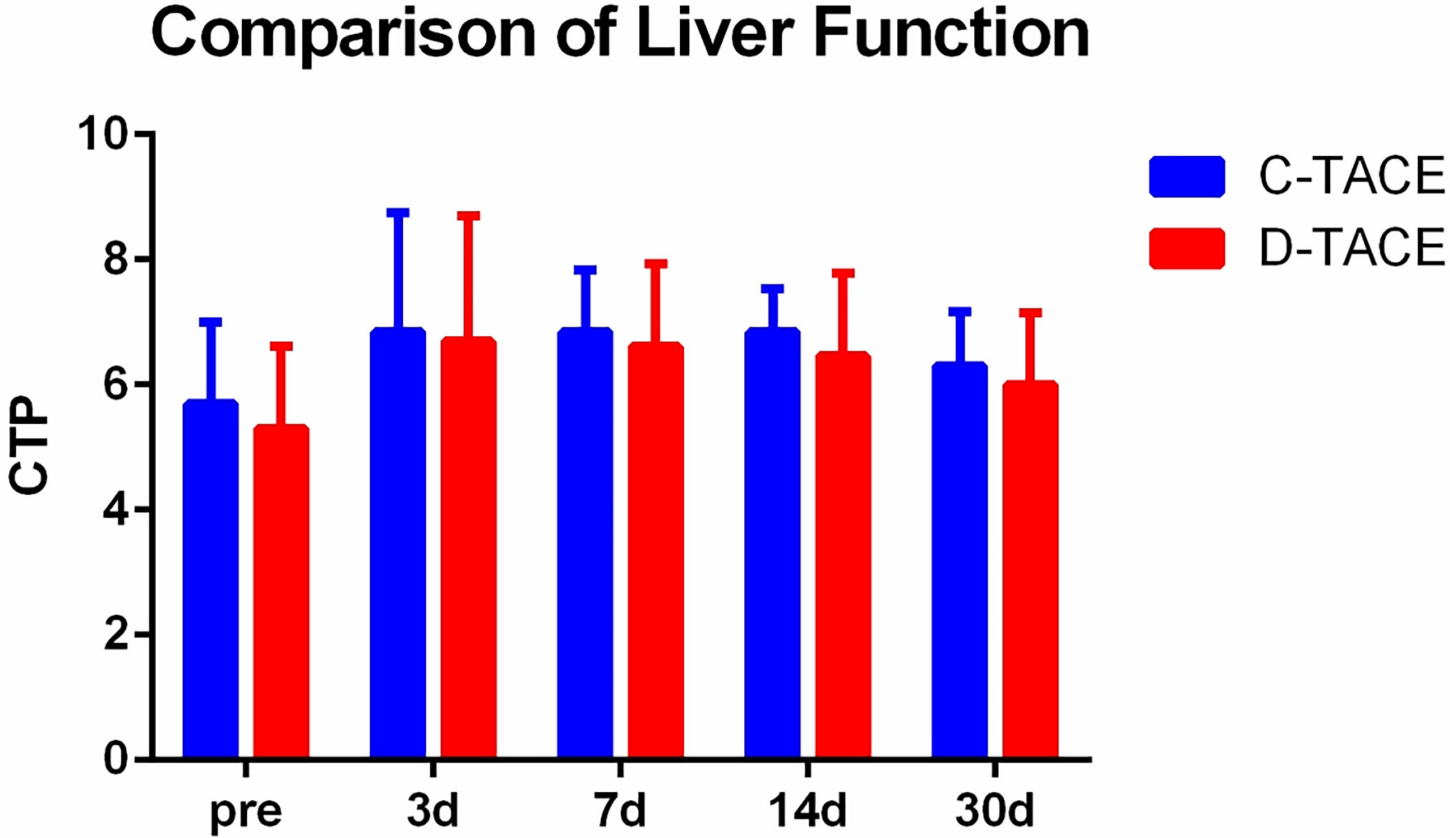
Comparison of liver function. There were no significant differences in Child-Pugh scores between the two groups before the procedures (p=0.47) or at 3 days (p=0.77), 7 days (p=0.66), 14 days (p=0.47), and 30 days (p=0.56) after the procedures.

The alanine aminotransferase (ALT), aspartate aminotransferase (AST) and serum total bilirubin (TBil) levels significantly increased at 3 days and 7 days after the procedures but returned to preoperative levels after 30 days (**Table 2**). There were no significant differences in Child-Pugh scores between the two groups before the procedures (p=0.47) or at 3 days (p=0.77), 7 days (p=0.66), 14 days (p=0.47), and 30 days (p=0.56) after the procedures (**Figure 3**).

**Table 2:** Liver function parameters before and 3 days, 7 days, 14 days, 30 days after interventional procedures.

		ALT (IU/L)	AST (IU/L)	TBil (μmol/L)	Child-Pugh score
C-TACE group	pre	48.6 ± 28.9	84.6 ± 43.7	30.6 ± 19.1	5.7 ± 1.3
	3d	100.3 ± 151.0	210.0 ± 356.5	37.6 ± 19.9	6.8 ± 1.9
	7d	132.6 ± 154.7	176.1 ± 135.0	48.5 ± 42.9	6.8 ± 1.0
	14d	90.7 ± 29.9	106.0 ± 58.0	103.6 ± 122.9	6.8 ± 0.7
	30d	34.5 ± 54.7	108.8 ± 56.8	53.3 ± 94.2	6.3 ± 0.9
D-TACE group	pre	53.8 ± 42.8	93.2 ± 54.5	21.0 ± 7.0	5.3 ± 1.3
	3d	451.3 ± 462.8	590.3 ± 428.9	47.9 ± 26.9	6.7 ± 2.0
	7d	200.8 ± 222.6	116.2 ± 67.6	51.6 ± 27.9	6.6 ± 1.3
	14d	121.7 ± 75.7	127.0 ± 121.4	123.1 ± 165.2	6.5 ± 1.3
	30d	47.7 ± 45.1	117.5 ± 145.8	51.5 ± 77.7	6.0 ± 1.2

ALT, alanine aminotransferase; AST, aspartate aminotransferase; TBil, serum total bilirubin.

No patient in either group had serious complications such as puncture bleeding, abdominal bleeding, tumor rupture, gastrointestinal bleeding, liver abscess, or bile aneurysm. Postoperative adverse reactions included postembolization syndrome, nausea, pain, fever and fatigue, all of which significantly improved after symptomatic treatment. Two patients with myocardial damage had chest discomfort and pain within 24 hours after the procedures in the D-TACE group treated with 80 mg (mixed with 2 bottles of DC beads) and 40 mg epirubicin (mixed with 1 bottle of DC beads), respectively. No abnormal electrocardiogram findings were observed, but the serum levels of AST, lactate dehydrogenase (LDH), and N-terminal pro B-type natriuretic peptide (NT-proBNP) within 24 hours after the procedures had transient increases. After oxygen therapy, sublingual nitroglycerin and other treatments, the indicators of myocardial damage gradually decreased after 3 days.

### Results of follow-up

**Figure 4:**
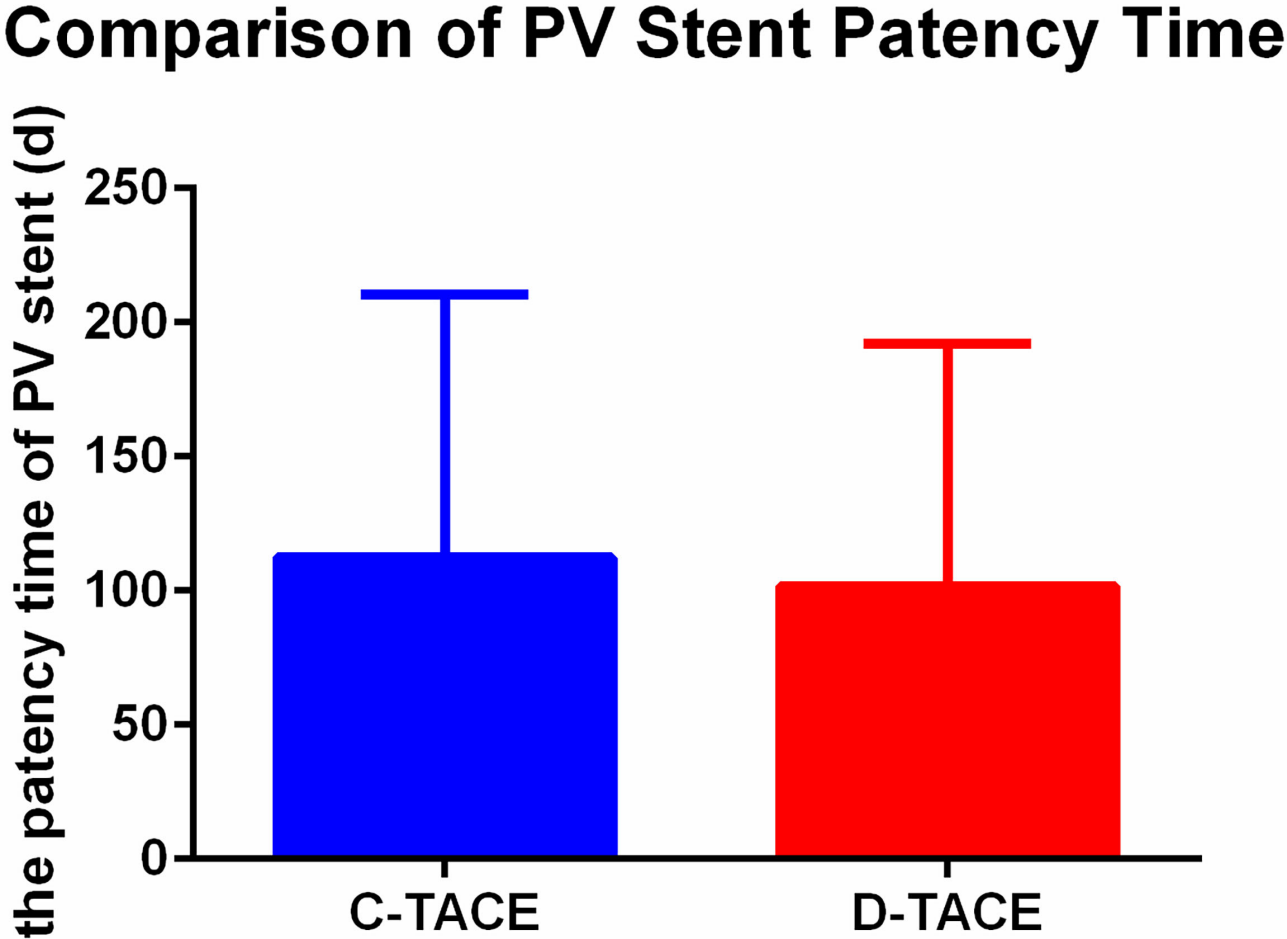
Comparison of PV stent patency time. The stent patency times of the two groups were 112.3 ± 98.2 days in the C-TACE group and 101.7 ± 90.4 days in the D-TACE group, and there was no significant difference between the groups (U = 84, p> 0.99).

The stent patency time of the two groups was 112.3 ± 98.2 days in the C-TACE group and 101.7 ± 90.4 days in the D-TACE group, and there was no significant difference between the groups (U = 84, p> 0.99) (**Figure 4**).

**Figure 5:**
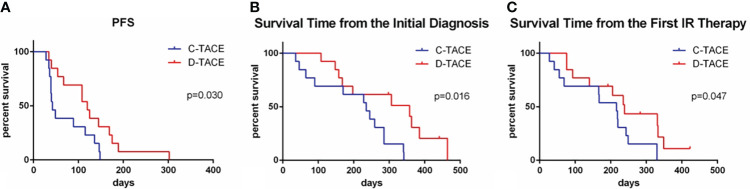
Comparison of PFS and OS in the two groups. The median progression-free survival times of the two groups were 42 and 120 days, respectively (p = 0.030). From the initial diagnosis, the overall survival times of the two groups were 235 days and 357 days (p=0.02). From the first interventional procedures, the overall survival times of the two groups were 216 days and 239 days, respectively (p=0.047).

According to the mRECIST criteria (6), the increased enhanced tumor tissue volume in CT or MRI images was used as the basis for evaluating disease progression. The median progression-free survival times of the two groups were 42 days and 120 days, respectively—significantly longer in the D-TACE group than in the C-TACE group (p = 0.03) (**Figure 5A**). By the end of the 5-year follow-up, 3 patients in the D-TACE group still survived. From the initial diagnosis, the overall survival times were 235 days and 357 days in the two groups (p=0.02) (**Figure 5B**). From the first interventional procedures, the overall survival times of the two groups were 216 days and 239 days, respectively (p=0.047) (**Figure 5C**). The difference was statistically significant: the D-TACE group was superior to the C-TACE group regarding both PFS and OS.

## Discussion

### Portal vein stents for HCC patients with MPVTT

This study demonstrated that endovascular implantation of a stent with a ^125^I seed strand combined with D-TACE is a safe and effective option for managing HCC patients with MPVTT. Portal vein thrombosis is an important factor in the prognosis of HCC patients. For unresectable cases including those with major vessel invasion, many community hospitals do not attempt active oncologic therapy. Recently, some studies have suggested that local treatment including radiotherapy will have survival benefits for these patients (7, 8). Other methods based on C-TACE combined with targeted drugs, radiation therapy, or thermal ablation also have been reported (9–11). Portal vein revascularization through endovascular stenting is also one of the ways to solve this difficult problem with improvement of liver parenchymal perfusion, relieving sequelae of portal hypertension and regaining of subsequent liver function (12). In this study, the mean stent portal vein pressure was 28.3 ± 11.2 cmH_2_O in the C-TACE group and 25.2 ± 12.3 cmH_2_O in the D-TACE group, which decreased to 23.6 ± 10.2 cmH2O and 20.2 ± 11.7 cmH_2_O, respectively, after the stent implantations, suggesting that implanting portal vein stents may decrease the portal vein pressure and reduce the risk of secondary gastrointestinal bleeding in patients with MPVTT.

### Impact of endovascular brachytherapy

Endovascular brachytherapy with ^125^I seeds can be continuously performed and has a long half-life (approximately 60.1 d). Close contact with the tumor tissue under the continuous emission of X-rays and γ-rays from the ^125^I seed can destroy the double-stranded DNA of tumor cells and inhibit the growth of tumor thrombi (13). Local irradiation can also inhibit vascular endothelial proliferation (14) and prolong the duration of stent patency. In this study, an appropriate amount of ^125^I seeds were packaged into 3F sterile sheaths to develop seed strains based on tumor thrombus length (measured by main portal vein angiography); then, the patients were synchronously implanted with a portal vein stent and the stent was expanded. The ^125^I seed strains were fixed to the tumor thrombus site, effectively preventing loss and displacement. Portal vein stent implantations were successfully completed in all the patients and the ^125^I seed strains were implanted, suggesting that this combined therapy method is highly feasible. All patients successfully completed the procedures under local anesthesia with good tolerance in this study.

The combined toxicity of endovascular brachytherapy and TACE needs attention. Some literatures have confirmed that the combination of radiation therapy and TACE does not cause significant adverse effects on liver function with the grade 3 toxicity rates ranged from 3.5% to 5.7% (15, 16). Other studies have shown (17) that compared with C-TACE, patients’ tolerance of D-TACE is better, with less-severe liver toxicity and fewer doxorubicin-related side effects. However, no study has reported the combination of endovascular brachytherapy and D-TACE in the treatment of HCC patients with MPVTT. The present study preliminarily explored the safety of this combination as well as the feasibility. Liver function was transiently abnormal but recovered gradually. The recovery of serum Tbil was slower than that of ALT and AST, which may be related to bile duct injury after embolism. The parameters of two patients with myocardial damage gradually decreased 2 to 3 days after oxygen therapy and nitroglycerin sublingual treatments. The epirubicin dosages of the two patients were 80 mg and 40 mg, respectively; based on patients’ body surface area, these were not overdoses, suggesting the possible cardiotoxicity of D-TACE when loaded with epirubicin or doxorubicin. Therefore, patient heart function needs to be monitored closely and treated promptly. Overall, the combination of the implantation of radioactive seed stents into the portal vein and D-TACE in the treatment of HCC patients with MPVTT is both feasible and safe.

### Comparison of C-TACE and D-TACE

D-TACE has been applied as a treatment for HCC for many years, and a number of clinical trials have demonstrated its safety (18) and effectiveness (4). A DC Bead^®^ is a drug-loaded microsphere that was approved in China in August 2014. The bead can be loaded with doxorubicin, epirubicin, or irinotecan. After embolism, the drugs can be continuously released with a certain amount of compressibility, which can effectively block the target vessel. One prospective randomized controlled study (17) compared C-TACE with D-TACE in the treatment of HCC and found that the complete remission rate, objective response rate and disease control rate in the D-TACE group were higher than those in the C-TACE group (27% vs 22%, 52% vs 44%, and 63% vs 52%, respectively) but without significant differences. However, the objective response rate in the D-TACE group was significantly higher than that of the C-TACE group among cases with Child-Pugh grade B, ECOG score 1, involvement of two lobes, and relapse.

A randomized controlled study (19) compared the effectiveness of simple microembolization (BB group) and the drug doxorubicin in microsphere embolization (LCB group) in patients with HCC and found that the response rates (RECIST criteria) of the BB group and LCB group at the first revisit were 5.9% and 6.0%, respectively. The median PFS values of the BB group and LCB group were 6.2 months and 2.8 months, respectively, which were not significantly different. The median overall survival of the BB and LCB groups were 19.6 months and 20.8 months, respectively, which were not significantly different. The embolization effects of ordinary microspheres and drug-loaded adriamycin microspheres on HCC were not significantly different. Drug-loaded adriamycin microspheres may not be able to improve the effectiveness of liver cancer embolization. The long-term efficacy of D-TACE needs further investigation (20). Therefore, we followed up these cases for 5 years, a length of time that not only allows assessing the feasibility and safety of the combined treatment method but also objectively evaluating the long-term efficacy of the combined treatment. In our study, the D-TACE group was superior to the C-TACE group regarding both PFS and OS. Compared with other studies, our patients had a relatively late disease course, showing that the combined treatment may have more advantages for patients with more severe disease.

Our study had some limitations. The sample size was relatively small. More cases are needed to reach more reliable results. All these patients were in the BCLC-C stage, and treatment with targeted drugs such as sorafenib may have yielded better results (21). However, due to economic constraints or concerns about the side effects of targeted drugs, these patients were not able to combine targeted drugs at the same time. Follow-up studies are also needed on patients who are being treated with combined targeted drug therapies.

In conclusion, the combination of the implantation of a radioactive seed stent to the portal vein and D-TACE in the treatment of HCC patients with MPVTT is both safe and feasible. This combination therapy may be helpful for survival benefits to patients with stage BCLC-C HCC.

## Data availability statement

The raw data supporting the conclusions of this article will be made available by the authors, without undue reservation.

## Ethics statement

The studies involving human participants were reviewed and approved by Ruijin Hospital Affiliated to Shanghai Jiao Tong University School of Medicine. The patients/participants provided their written informed consent to participate in this study.

## Author contributions

ZWu, WH and JGo contributed to conception and design of the study. JGu and QW organized the database. JL and QL performed the statistical analysis. WH and JGo wrote the first draft of the manuscript. JGu, XD and ZWa wrote sections of the manuscript. All authors contributed to the article and approved the submitted version.

## Funding

This work was supported by National Natural Science Foundation of China (Grant No. 62173223), Shanghai Key Specialty Construction Project (No. ZK2019A02) and Shanghai Municipal Key Clinical Specialty (grant numbers shslczdzk06002, shslczdzk07002)

## Conflict of interest

The authors declare that the research was conducted in the absence of any commercial or financial relationships that could be construed as a potential conflict of interest.

## Publisher’s note

All claims expressed in this article are solely those of the authors and do not necessarily represent those of their affiliated organizations, or those of the publisher, the editors and the reviewers. Any product that may be evaluated in this article, or claim that may be made by its manufacturer, is not guaranteed or endorsed by the publisher.
